# Quantitative proteomics and network analysis of SSA1 and SSB1 deletion mutants reveals robustness of chaperone HSP70 network in *Saccharomyces cerevisiae*

**DOI:** 10.1002/pmic.201400527

**Published:** 2015-04-10

**Authors:** Andrew F. Jarnuczak, Claire E. Eyers, Jean‐Marc Schwartz, Christopher M. Grant, Simon J. Hubbard

**Affiliations:** ^1^ Faculty of Life SciencesMichael Smith BuildingManchesterUK; ^2^ Centre for Proteome ResearchDepartment of BiochemistryInstitute of Integrative BiologyUniversity of LiverpoolLiverpoolUK

**Keywords:** Betweenness centrality, Chaperones, Protein interaction networks, SILAC, Systems biology, Quantitative proteomics

## Abstract

Molecular chaperones play an important role in protein homeostasis and the cellular response to stress. In particular, the HSP70 chaperones in yeast mediate a large volume of protein folding through transient associations with their substrates. This chaperone interaction network can be disturbed by various perturbations, such as environmental stress or a gene deletion. Here, we consider deletions of two major chaperone proteins, *SSA1* and *SSB1*, from the chaperone network in *Sacchromyces cerevisiae*. We employ a SILAC‐based approach to examine changes in global and local protein abundance and rationalise our results via network analysis and graph theoretical approaches. Although the deletions result in an overall increase in intracellular protein content, correlated with an increase in cell size, this is not matched by substantial changes in individual protein concentrations. Despite the phenotypic robustness to deletion of these major hub proteins, it cannot be simply explained by the presence of paralogues. Instead, network analysis and a theoretical consideration of folding workload suggest that the robustness to perturbation is a product of the overall network structure. This highlights how quantitative proteomics and systems modelling can be used to rationalise emergent network properties, and how the HSP70 system can accommodate the loss of major hubs.

**Abbreviations**
BC: betweenness centrality
FDR: false discovery rate
LOESS: locally weighted regression
MA: mean versus average

## 1 Introduction

Molecular chaperones, historically known as heat shock proteins, play important roles in the cellular stress response, also contributing to cellular protein homeostasis under normal conditions through a variety of mechanisms 1, 2, 3, 4. Chaperone activity is underlined by their ability to form interactions with client proteins, as well as other proteins, e.g. co‐factors or ribosomal proteins, in order to mediate their correct folding and subsequent trafficking. Those interactions give rise to a complex protein–protein interaction network that can be easily graphically visualised and provides a conceptual map for interpreting their integrated global function. For example, the yeast chaperone network contains 63 well‐known chaperone proteins, many of which have been extensively studied for their biochemical properties, molecular mechanisms of protein folding, individual role in stress response and molecular aspects of chaperone‐mediated proteostasis. However, a holistic, system‐level understanding of the network where all the elements (chaperones and substrates) are considered simultaneously has yet to be defined. Such a model would clearly be highly informative. As a prerequisite to building such a model, the network of chaperone‐substrate interactions should be defined, to provide a biological framework in which the emergent properties of the cell, such as protein‐folding homeostasis, can be explained 5. A system of 20 or 30 proteins organised in a network is capable of performing functions that an uncoupled collection of proteins cannot; what might be difficult to explain in the context of single elements becomes ‘obvious’ in a network 6.

Seminal work by Gong et al. 7 using tandem affinity purification followed by MS analysis was the first large scale, targeted identification of protein complexes within *Saccharomyces cerevisiae* chaperone networks and delineated what can be dubbed as the ‘yeast chaperome’. The chaperome defined by Gong et al. 7 contains 63 chaperone proteins and is a system of sub‐networks that can be divided into two principal modules: functionally non‐selective chaperones (typically > 200 interactions with non‐chaperone proteins) and functionally specific chaperones (fewer than 200 non‐chaperone interactions). This classification is based on the number of interacting proteins and is the simplest strategy aiming to interpret the network by finding common parameters (or attributes) of chaperones. More fine‐grained chaperome functional modules have also been identified based on shared interactions 7 or by clustering chaperones into groups based on the target proteins with which they interact 8, 9. This can define topological clusters, as has been successfully used by Bogumil et al. 9 to discover distinctive chaperome community structure linked to evolutionary properties.

While the entire chaperone network is involved in the many biological processes relating to cellular protein homeostasis 10, a sub‐network of HSP70 molecular chaperones are thought to be chiefly responsible for mediating de novo protein folding through a mechanism involving cycles of binding and release of substrate proteins, triggered by ATP and HSP40 cofactors 11. They also have known functions in protein translocation across biological membranes and dissolution of aggregates 12. Nascent and newly synthesized polypeptides are bound by HSP70, initially stabilising the unfolded state; upon subsequent release into solution they then reach their native folded state 9. A loss of part of this folding mechanism could potentially result in increased numbers of misfolded, unfolded or aggregated proteins, particularly for proteins that exclusively interact with just one HSP70 chaperone. The misfolded proteins are then destined for a number of possible outcomes: vacuolar and nonvacuolar pathways of degradation (resulting in measureable ‘protein down‐regulation’), or aggregation resulting in apparent protein ‘up regulation’. Alternatively, it is possible that other mechanisms take over the responsibility of folding these proteins (network redundancy, alternative chaperonin and HSP90‐mediated folding etc.) and no major phenotypic changes are visible.

**Figure 1:**
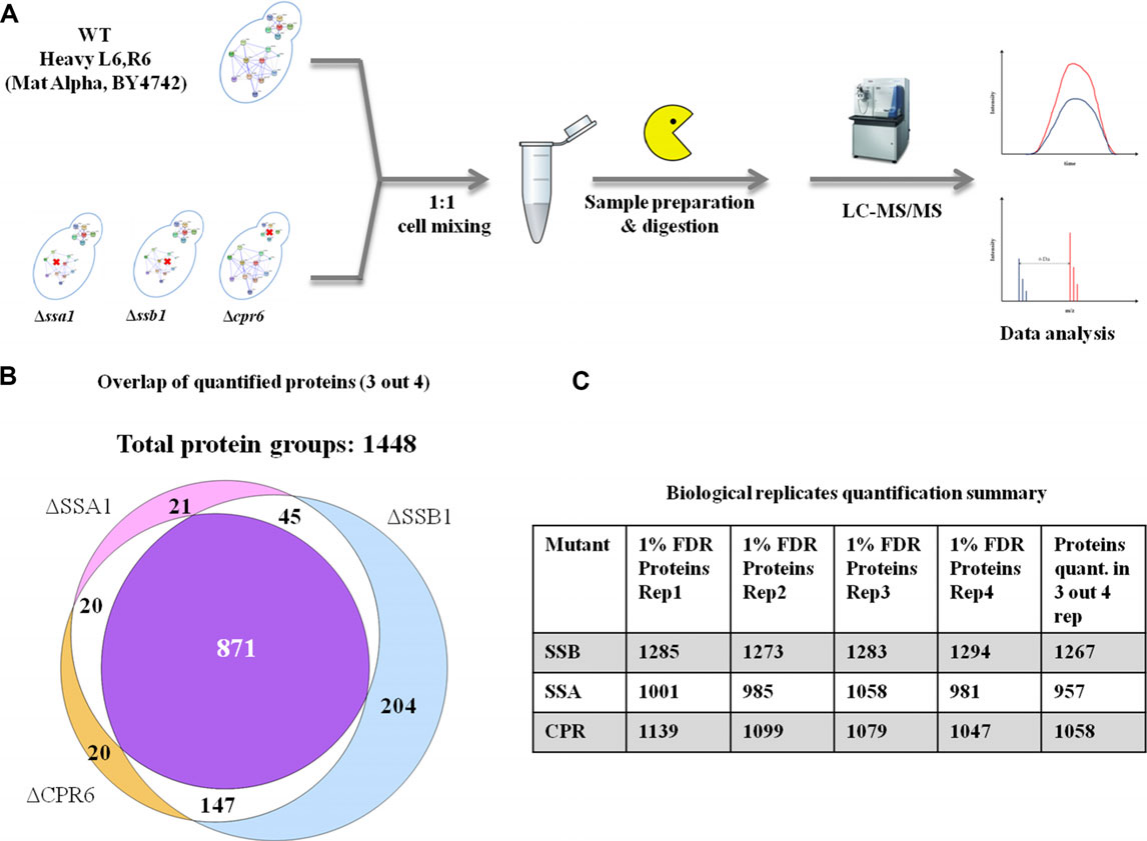
Overview of the experimental workflow and MS‐based quantification of yeast proteins. (A) Four yeast gene deletion mutants were selected for SILAC‐based proteome quantification. Cells were grown to midexponential phase in batch culture, harvested, and processed for LC‐MS analysis. Peptide data was acquired on an LTQ Orbitrap Velos and raw spectra were quantitatively analyzed with MaxQuant software. All experiments were performed in biological quadruplicates. (B) Only proteins identified and quantified in three out of four biological replicates were included in further analysis. The Venn diagram shows protein overlap for these quantifications between different mutants. (C) The actual numbers of proteins quantified in each biological replicate and final number of proteins used for each mutant are shown (i.e. proteins quantified in at least 3 of 4 biological replicates within each mutant sample). All reported identifications were at 1% peptide and protein FDR.

While protein interactions define the chaperome at the molecular systems level, they provide only a static definition of the network under normal growth conditions; we also wish to characterise how the system changes under perturbation (e.g. mutations, gene deletions, environmental stress or protein up‐regulation). Here, we focus on gene deletions in *S. cerevisiae*, to study how loss of a major chaperone protein can impact global cellular protein abundance and understand the attendant changes through network analysis. Our overall experimental design is shown in Fig. 1. We selected two mutants from the HSP70 subfamily of cytosolic chaperones: heat shock protein *SSA1* (YAL005C) and heat shock protein *SSB1* (YDL229W) and compared them to a wild‐type strain (WT). As an additional control, we also considered the proteome changes in the yeast strain lacking peptidyl‐prolyl cis‐trans isomerise (*CPR6*, YLR216C) (Fig. 1A). This gene is considered as a co‐chaperone of HSP90 (to which it binds and can regulate activity), although it was not considered by Gong et al. 7 and is not an HSP70 family chaperone; this strain is used here strictly for the cell size/total protein content correlation comparison.

From the network perspective *SSB1* and *SSA1* are obvious targets for analysis as they are the two biggest hub proteins in the chaperone network, with 3269 and 2489 client‐protein links, respectively, as well as interactions observed with over 40 other chaperones 7. They are also highly abundant in unstressed yeast cells, estimated at 8178 ppm for *SSA1* and 2320 ppm for *SSB1* (in the top 5% of yeast proteins by abundance according to PaxDB) 13. The two proteins are highly homologous to each other (63% sequence identity) and also to their closely related paralogues *SSA2* and *SSB2*; SSA1/SSA2 sequence identity stands at 98%, while SSB1/SSB2 is 99% 14.

The HSP70 chaperone family, including SSA1/SSA2 and SSB1/SSB2 proteins, has been extensively studied in terms of evolutionary similarity 14, 15, 16 and functional similarity/redundancy via knock‐out models 17, 18, 19, 20, 21, 22, 23, 24, 25, 26, 27. For example, Craig et al. 22 characterised members of the SSA family in yeast and reported no phenotypic effects when a single gene, either *SSA1* or *SSA2* was deleted. The deletant strains grew at similar rates to the wild‐type and did not show any obvious phenotypic differences under heat shock (defined in the original publication as the ability to withstand exposure to 52°C after a 50‐min pre‐treatment at 37°C). A double (*SSA1/SSA2*) mutant was growth retarded at 30°C and inviable at 37°C. However, despite these similarities, *SSA1* and *SSA2* are differentially regulated 26; SSA1 expression is highly increased at 39°C while SSA2 transcript abundance increases only marginally in higher temperatures. A number of other studies have also pointed to functional specificity, as reviewed by Kabani and Martineau 28. Furthermore, when grown on a non‐fermentable carbon source and at 37°C, the *SSA1* mutant strain is inviable whilst cells deleted for *SSA2* grow normally 27. Collectively, these results suggest both phenotypic redundancy between *SSA1* and *SSA2* and different molecular and regulatory properties.

*SSB1* and *SSB2* gene function has also been well characterised 14, 23, 29. Although SSB proteins display over 50% identity with SSA chaperones they are functionally distinct, as demonstrated by a series of experiments 27. Their activity (unlike SSA proteins) is also mainly confined to the ribosome, where they function to facilitate translation and proper protein folding 23, 30 and their transcription is coupled with ribosomal proteins 20. Another notable difference between *SSA* and *SSB* families is that SSA1 and SSA2 proteins have been shown to depolymerise clathrin vesicles in vitro, while SSB proteins lack this ability 31. Similarly to *SSA*, no obvious phenotypic effects of a single gene deletion were observed with *SSB1/SSB2* mutants whilst the double *SSB1/SSB2* mutation showed very poor growth at 19°C 23.

**Figure 2:**
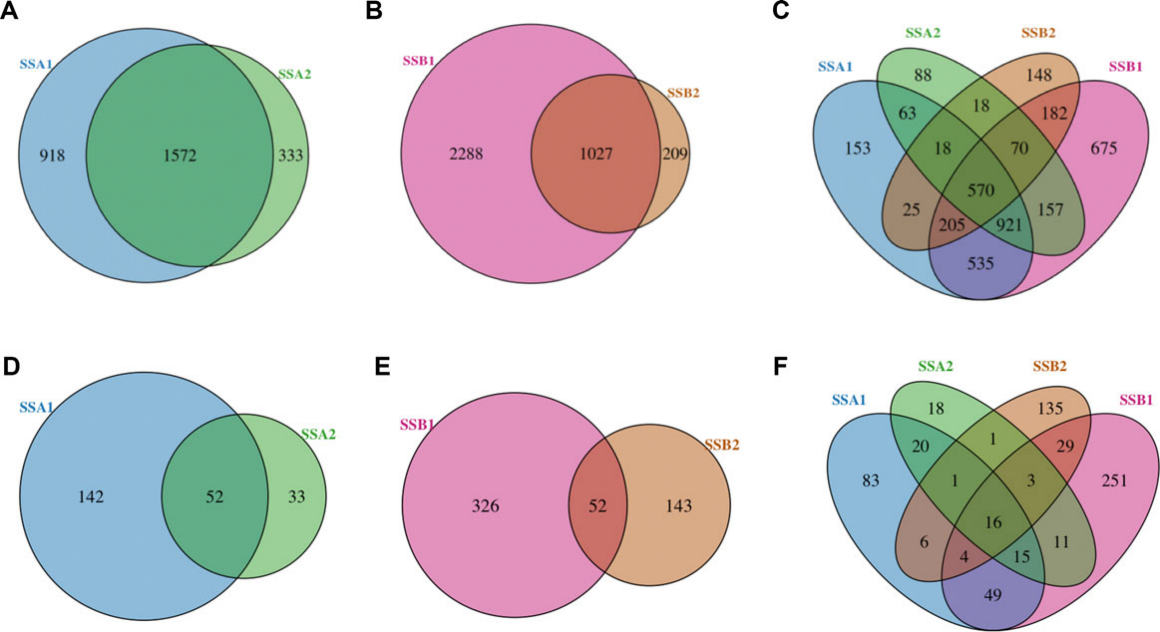
Overlap between protein interaction partners for SSA1, SSA2, SSB1, and SSB2 members of the HSP70 chaperone family. The Venn diagrams shown in panels (A)–(C) show the number of substrates that interact with each given HSP70 protein according to 7 from: (A) the SSA family, (B) the SSB family, and (C) all four proteins. Substantial numbers of substrates are unique to the individual chaperones. Panels (D)–(F) show the same data, restricted to a filtered, high‐quality subset of the chaperone network 32, constituted by 3649 chaperone‐substrate interactions.

Although the phenotypic robustness of the HSP70 family is fairly well established, these observations usually relate to doubling times or growth assays and are rationalised largely in term of sequence similarity. However, these studies have not formally considered the underlying network response to a deletion at the molecular level. Although the SSA/SSB pairs share high sequence identity, their sets of interaction partners are quite different, as shown in Fig. 2, where each chaperone has its own unique set of interactors. For example, based on the reported interactome 7, 918 substrate proteins interact exclusively with SSA1 and not SSA2 (Fig. 2A) and 2288 substrates interact only with SSB1 and not with SSB2 (Fig. 2B). Similarly, each of the four HSP70 chaperones considered has unique interactors not associated with the others (Fig. 2C). This relatively modest overlap between paralogue pairs suggests that simple sequence identity alone is insufficient to explain the phenotypes observed. We note this modest overlap is further reduced when the network is filtered and limited to 3649 chaperone‐substrate pairs present in multiple datasets 32, shown in Fig. 2D–F, a high quality dataset designed to reduce the level of false positives.

Hence, to obtain a global molecular view of the chaperone system and its response to perturbations in yeast grown under standard batch condition, we quantified proteome changes in *SSA1* and *SSB1* mutants using a SILAC approach 33. Protein content ‘per cell’ was considered in addition to changes in individual protein concentrations. Our results show that despite being major chaperome hubs, the abundance of relatively few substrates appears to be affected. The intracellular chaperome network is apparently remarkably robust to deletions and able to maintain general protein homeostasis in order to accommodate the loss of the two HSP70s. Despite this, phenotypic changes are observed in terms of cell size, which are discussed in terms of total protein abundance and the attendant implications for data normalisation strategies employed to deal with SILAC data of this nature. Finally, we attempt to rationalise the observed experimental changes in terms of network theory, using in silico graph theoretical approaches and considerations of folding workload. The theory lends good support to the experimental observations; namely, that the network compensates through increases in node workload as a mechanism to sustain stability in light of deletions of its two biggest hub proteins.

## 2 Materials and methods

### 2.1 Yeast sample preparation and SILAC labelling

The wild‐type strain BY4742 (*Mat α, his3Δ1, leu2Δ0, lys2Δ0, ura3Δ0*) and its isogenic derivatives deleted for *SSA1* (YAL005C), *SSB1* (YDL229W), and *CPR6* (YLR216C) were purchased from the Thermo Yeast Knockout (YKO) Collection. Strains were grown in Yeast Nitrogen Base (YNB) medium (20 g/L glucose) supplemented with amino acids arginine (10 mL/L), leucine (20 mL/L), uracil (10 mL/L), histidine (3 mL/L) and lysine (10 mL/L) on a shaker (30°C, 300 rpm) in biological quadruplicates. The WT strain was SILAC‐labelled by growing in YNB medium supplemented with ‘light’ amino acids leucine (20 mL/L), uracil (10 mL/L), histidine (3 mL/L) and ‘heavy’ ^13^C_6_‐lysine (10 mL/L) and ^13^C_6_‐arginine (10 mL/L). Cells were harvested in exponential growth phase at OD_600_ = 2 (± 10%) by centrifugation at 4000 rpm for 10 min at 4°C, aliquoted and stored at –80°C until further use. Cells were counted for each biological replicate using a Cellometer cell counter (Cellometer AUTOM10 by Nexcelom. http://www.nexcelom.com).

### 2.2 Protein extraction and digestion

Cell pellet was re‐suspended in 800 μL of ice‐cold 50 mM NH_4_HCO_3_ (with added Roche Complete mini protease inhibitor 1 tablet/10 mL of buffer) and transferred to a 2 mL screw cap vial for bead beating. Two hundred fifty microliters of acid washed glass beads was added and the pellet undergone 15 × 30 s cycles of bead‐beating with 1 min break in between each cycle in order to break the cell wall. The resulting lysate was centrifuged for 10 min at 16 000 rpm (4°C) and the supernatant removed and stored on ice. In order to maximise protein recovery an additional 250 μL 50 mM NH_4_HCO_3_ was added and the remaining pellet was re‐suspended by vortexing. A hot needle was used to pierce a hole in the bottom of the vial that was immediately placed inside a 2 mL Eppendorf tube and spun down for 10 min at 16 000 rpm (4°C). The wash and cell debris was collected as flow through and combined with the supernatant from previous step. The exact total volume of the combined fractions was determined and the volume corresponding to 30 million cells was aliquoted into low bind Eppendorf tubes. This was stored at –80°C until further use. Appropriate volume of SILAC‐labelled WT standard was then mixed with the knock‐out strains so that they contained equal amounts of cells (30 million each). The combined samples were diluted to a final volume of 320 μL with 25 mM ammonium bicarbonate. Proteins were denatured with RapiGest™ detergent (20 μL of 1% w/v, 80°C for 10 min), reduced, and alkylated using 60 mM dithiothreitol (60°C, 400 rpm shaking for 10 min) and 180 mM iodoacetamide (incubation at room temperature in the dark for 30 min). Twenty microliters aliquot of 0.2 μg/μL trypsin (Sigma, Poole, UK, proteomics grade) solution in 50 mM acetic acid was added and sample incubated at 37°C, 400 rpm shaking for 4.5 h followed by further addition of trypsin and overnight incubation. To quench the digestion reaction and hydrolyse the RapiGest detergent 5 μL of 50% TFA was added to the reaction mixture. The detergent was precipitated at 37°C, 400 rpm shaking for up to 2 h. Further volume of acetonitrile/water (2:1) was added and samples were incubated at 4°C for at least 2 h. RapiGest was then removed by centrifugation and samples stored at 4°C and used within a week.

### 2.3 LC‐MS/MS analysis

Tryptic digests (2.5 μL, corresponding to roughly 500 ng of protein) were loaded onto a C‐18 trap column at a flow rate of 5 μL/min. Peptides were separated on a 5 μm × 50‐mm C‐18 ACQUITY analytical column using a gradient of 10 to 60% MeCN over 270 min at a flow rate of 5 μL/min. The column was coupled to a LTQ Orbitrap Velos via a nanoelectrospray source. LTQ Orbitrap was operated in data‐dependent mode in the scan range of *m/z* 300–1600 with survey scans acquired in the FTMS at a resolution of 60 000 at *m/z* 400. Twenty most intense precursor ions with charge ≥ 2+ from the survey scan were selected and fragmented by CID. Ions selected for fragmentation were excluded for 60 s before being considered for fragmentation again.

### 2.4 MaxQuant data processing and bioinformatics analysis

The resulting raw files were processed with MaxQuant software 34 (version 1.4) according to an earlier described protocol 35. Briefly, peptides and proteins were identified from tandem MS spectra using the Andromeda 36 search engine (UniProt S. *cerevisiae* database, release: 2013_08). The search parameters relating to identification were set as follows: cleaving enzyme trypsin; fixed modifications: carbamidomethyl (cysteine); variable modifications: deamidation (asparagine, glutamine), methionine oxidation and N‐terminal acetylation. Mass shift of +6 Da was selected for arginine and lysine as SILAC heavy labels and ‘filter labeled amino acids’ option in ‘identification’ window was de‐selected. Up to two missed cleavages were allowed. Instrument type was set to Orbitrap and peptide mass tolerance was set to 20 ppm while MS/MS tolerance was set to 0.5 Da. The false discovery rate (FDR) for peptide spectral matches was set to 1%. Protein level FDR was also set to 1% and calculated based on a reverse decoy database search. Matching between runs was enabled with a 1 min match time window and 20 min retention time alignment window. The re‐quantify option was left de‐selected. Matching was only performed between biological replicates within each experiment. All other MaxQuant parameters were left as default.

For relative protein quantification Raw Heavy and Light Intensities reported by MaxQuant were used. Those were obtained only for unmodified peptides and proteins were required to have at least one unique or ‘razor’ peptide but to be present in at least three out of four biological replicates. This in effect resulted in the majority of quantified proteins having two or more peptides. The final reported protein SILAC ratios are the average of replicate measurements.

The Raw Intensities were further processed with R 37 and DanteR package 38. Intensities were log2 transformed and for normalisation, a linear regression to the replicates’ mean was applied to centre the distributions. The significance of any given fold changes was calculated using a ANOVA 39 test at a significance level of 0.05. Multiple testing p‐value correction was applied using the Benjamini & Hochberg false discovery rate method 40 in DanteR package 38.

### 2.5 Network generation and topological parameters analyses

The original network of chaperone interactions in *S. cerevisiae* was obtained from Gong et al. 7. Mutant networks were generated by removing the deleted node and all attendant links. This often resulted in a number of unconnected components (i.e. nodes that were originally interacting solely with the deleted node). In ∆SSA1 there were 64 such nodes (2.5% of all SSA1 interactors) and in ∆SSB1 269 nodes (8.1%). These nodes were retained and included in the analysis. All network edges were treated as undirected and had the same weight. This resulted in three principal networks; WT with 4403 nodes and 21946 undirected edges. ∆SSA1 network with 4402 nodes, and 19373 edges, and the ∆SSB1 network with 4402 nodes and 18585 edges. Network and node attributes were calculated within Cytoscape 41 using the NetworkAnalyser 42 plug‐in. The calculated parameters together with their definitions are listed below:

1. Node degree; degree of node *n* is the number of edges incident to *n*. Node degree distribution shows the number of nodes with degree *k = 1,2…*
2. Node neighbourhood connectivity; while node connectivity is the total number of its neighbours, neighbourhood connectivity of node *n* is the average connectivity of all neighbours of *n*.
3. Average clustering coefficient; the clustering coefficient is a measure to what extent nodes in a network tend to cluster together. It also reflects the probability that two randomly selected nodes are linked. The clustering coefficient is defined as $C_{n}=2e_{n}/\left(k_{n}\left(k_{n}-1\right)\right),$ where $k_{n}$ is number of neighbours of *n* and $e_{n}$ is the number of connected pairs between all neighbours of *n*.
4. Betweenness centrality (BC) is an approximation of how central a node is in the network and what influence it has over the transfer of information through the network. In the context of a chaperone network it indicates the workload placed on given chaperone (see results section for detailed explanation). BC is defined by the following equation:
  BC(p)=∑s,t∈Vp≠s≠tσst(p)σst(N−1)(N−2)2

where *V* is the set of all nodes (proteins) in the network, *s* and *t* are proteins in the network, $\sigma_{st}$ is the number of shortest paths between *s* and *t*, $\sigma_{st}\left(p\right)$ is the number of shortest paths that pass through *p* and N is the number of connected proteins in the network component. A more detailed description of network parameters together with comprehensive references to literature is also available from 42 and on the web at http://med.bioinf.mpi‐inf.mpg.de/netanalyzer/help/2.7/

Subsequent analysis and visualisation of node attributes was done in R 37. Briefly, node attribute distribution plots were generated by plotting the average value of all nodes at a given degree. Statistical comparison of average attribute distributions was done by comparing their empirical cumulative distribution functions with a two‐sided Kolmogorov–Smirnov test 43. The K‐S statistic assesses the maximum difference between two empirical cumulative distribution functions giving the maximum absolute distance between the two (*D*‐value) and a *p*‐value indicating whether they are equal. Finally, network visualisation diagrams were prepared in Cytoscape.

### 2.6 Network workload calculations

Following previous studies, chaperone workload was estimated as the sum of folding fluxes attributed to each chaperone. The folding flux (*F_c_*), of a chaperone (*c*) in terms of molecules per min per cell, is calculated over all *n* substrates as follows:

$$F_{c}=\sum_{i=1}^{n}k_{syn,i}$$

where *k_syn_* is calculated from known protein concentrations and protein degradation rates,

$$k_{syn,i}=cpc_{i}\;\times \;k_{deg,i}$$

assuming steady state where protein synthesis and degradation rates are equal ($\frac{dCPC_{i}}{dt}=0$). To reduce bias from potential false positives we performed these calculations with the high‐quality subset of 3649 interactions defined previously 32 rather than the full network. The substrate flux values where then divided equally pro rata among each chaperone. Any missing *k_deg_* values where replaced by the geometric mean across the entire turnover dataset; this was to ensure the mean *k_deg_* of the dataset remained unchanged. Protein concentrations were taken from PaxDB 13, using those reported from the de Godoy SILAC dataset 44.

## 3. Results and discussion

### 3.1 Quantification of protein content in yeast chaperone mutants (unnormalised SILAC intensities)

The *Saccharomyces* wild‐type strain was metabolically labelled with L‐arginine^13^C_6_ (Arg6) and L‐lysine^13^C_6_ (Lys6) to create a heavy reference sample for comparison with all the mutant strains. After establishing good labelling efficiency (near 100% for lysine peptides and above 90% for arginine peptides) verified in a separate experiment (Supporting Information Fig. 1) the heavy and light samples were mixed in equivalent cell ratios. Peptide data were acquired on an Orbitrap Velos in biological quadruplicates. Protein identification and quantification was done with MaxQuant software 34 and downstream data analysis was performed with R 37 and danteR package 38. A total of 1448 proteins were identified and quantified using MaxQuant (at 1% PSM and 1% protein FDR, see Experimental methods). To obtain the best quantitative results and improve performance of the statistical analysis, only proteins quantified in at least three out of four biological replicates (MS1 signal present for either light or heavy peptide) were selected for further analysis and are reported here (Fig. 1B, C); this amounted to 1267 proteins in the *ΔSSB1* mutant, 957 proteins in the *ΔSSA1* mutant and 1058 proteins in the *ΔCPR6*  mutant. Considering the strict filtering criteria, a good overlap of proteins was observed across all samples and a core of 871 proteins was quantified in at least 12 experiments (see Fig. 1B).

**Figure 3:**
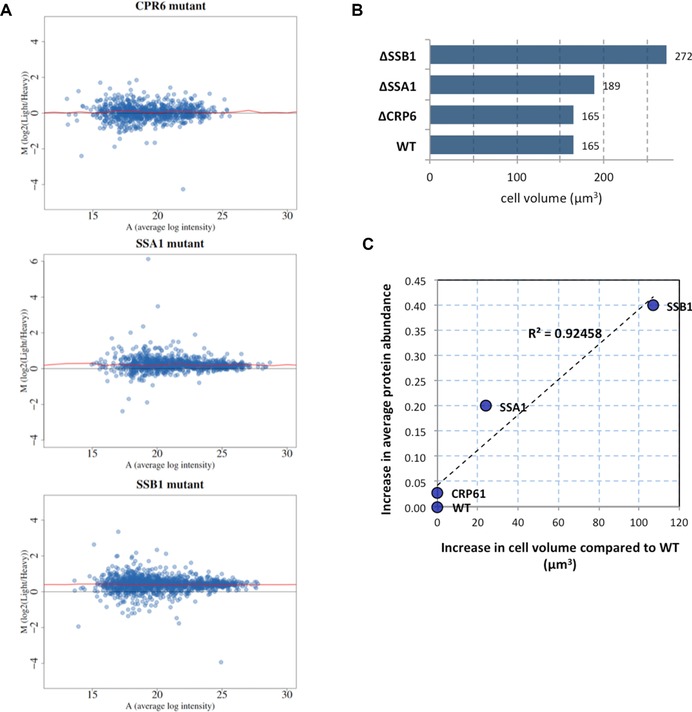
Protein abundance changes in chaperone mutants and cell size effects. (A) Mean versus average (MA) plots of protein abundance levels derived from MaxQuant raw intensity values in the three different mutant yeast strains. MA plots depict the average protein intensity (*y* axis) against the calculated log_2_ (L/H) protein ratio (*x*‐axis) across four biological replicates. Points below *y* = 0 represent proteins upregulated in wild‐type yeast and points above *y* = 0 are proteins upregulated in a mutant strain. The red line presents a fitted nonlinear, locally weighted regression (LOESS) curve, indicates an absence of abundance‐dependent bias, though there is a systematic global shift (i.e. increase in protein content per cell) in the *SSB1* and to a lesser extent in *SSA1* mutant; (B) bar chart of cell volumes of yeast mutants based on measurements taken from *S. cerevisiae* morphological database, and (C), relationship between change in cell volume for individual mutants and the median fold change in measured heavy/light protein ratios in mutant yeast strains. The median and average measurements of fold changes gave similar results (data not shown).

Mean versus average (MA) plots illustrating the distribution of unnormalised protein fold changes and their average MS1 signal intensity in the three mutant strains are shown in Fig. 3A, highlighting proteins that are considered to be up‐ or down‐regulated in mutant strains. The fold change depicted in these plots is an average of unnormalised replicate measurements (log2(Light Intensity/Heavy Intensity)). The MA plots support assessment of both global expression bias and local, non‐linear intensity‐dependent bias. The systematic biases can be introduced as an artefact of sample preparation or MS data acquisition but could also be due to a real biological difference. Here, the unnormalised protein ratios show a generally symmetrical distribution with no evident abundance‐dependent bias, as indicated by a fairly uniform locally weighted regression (LOESS) curve running parallel to the axis. However, the LOESS curve is shifted up and away from the x‐axis in the two chaperone mutant strains (*SSA1* and *SSB1*) pointing to a global bias in protein abundance. Since the cells were mixed in a 1:1 ratio, such a shift suggests an increase in the total protein content in the mutant strains compared to the wild type. In contrast, negative values (*M* < 0) would be indicative of a decrease in the overall protein amount in mutant samples. As would be expected the cell‐size control *ΔCRP6* strain did not exhibit any variance in total protein content.

To estimate the differences in overall protein concentration, the median fold change for each sample was calculated. *ΔSSA1* and *ΔSSB1* showed a shift from 0 with median fold change 0.2 and 0.4, respectively. *ΔCRP6* had a fold change less than 0.03 indicating no major differences in protein concentration.

Next, the cell size of the mutant strains was considered (Fig. 3B). Cell size is the most basic feature of yeast morphology and can be measured using various methods. Here, we took values for each of the mutant strains under consideration from The *Saccharomyces cerevisiae* Morphological Database 45 that contains a comprehensive set of parameters describing cell morphology derived from micrographs of budding yeast mutants identical to the haploid MAT *α* strains obtained from EUROSCARF and used in the current work. We hypothesised that overall cell volume and protein content are likely to be coupled. As a simple rule of thumb, yeast cells have a roughly oblate spheroid shape described by two principal dimensions: an equatorial (*r*_1_) and a polar (*r*_2_) diameter, their volume (*V*) can be calculated from the following equation:

$$V=\frac{4}{3}\pi r_{1}r_{2}^{2}$$

Unsurprisingly, there is a correlation between cell volume and total amount of protein. When we consider the difference in volume and the difference in average protein fold change per cell (un‐normalised values as shown in Fig. 3C) an increase in cell volume results in global increase in protein content (average protein fold change increases). Interestingly, while the majority of proteins follow this trend and are up‐regulated in mutant strains of *S. cerevisiae* with larger cells, a small number of outliers exhibit a noticeable decrease in expression levels (Supporting Information Fig. 2). For example, in *ΔSSA1*, long‐chain‐fatty‐acid‐CoA ligase 4 (LCF4; P47912) and UPF0743 protein YCR087C‐A (YC16; P37263) are significantly down‐regulated (ANOVA *p*‐value < 0.05) with fold changes –1.9 and –0.3, respectively (Supporting Information Fig. 2). While in *ΔSSB1* four of the SSB1 targets (HSP12, LSM6, ATPG, and PSA2) as well as five other proteins showed statistically significant decreased expression (Supporting Information Fig. 2).

### 3.2 Identification of significant protein concentration changes in ΔSSA1 and ΔSSB1 strains (normalised SILAC intensities)

**Figure 4:**
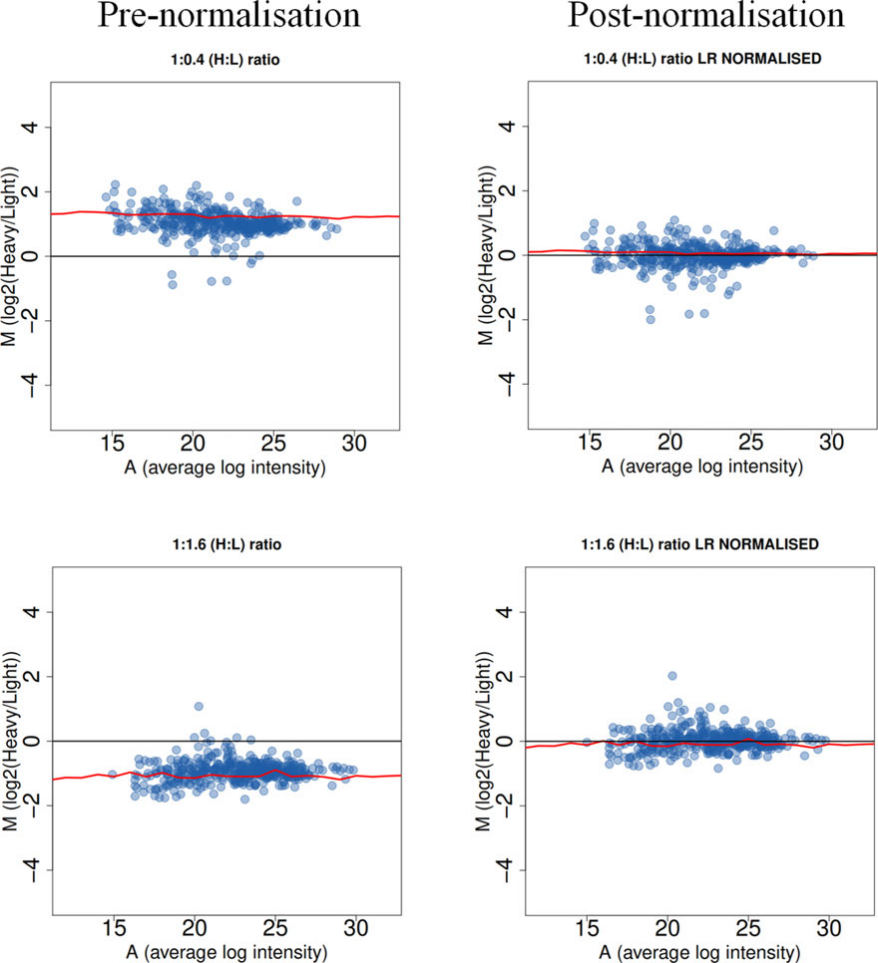
MA plots showing the effect of linear regression normalisation on SILAC standards of unequal cell (protein) spike in. Two standards were prepared by mixing ‘heavy’ and ‘light’ yeast samples in unequal ratios (1:0.4 and 1:1.6). After acquiring data in technical triplicates, average log_2_(H/L) ratios are plotted against average intensity, using the raw signal intensity from MaxQuant. Linear regression normalisation is performed by minimising the sum of squares of the errors (residuals) of the points from predicted straight line through data points (linear least squares fitting). Mean intensity of all datasets (replicate measurements) was used as reference set. Normalisation procedure forces the median of each protein ratio distribution to a log_2_ FC = 0, corresponding to a ratio of 1:1 protein on column.

Next, we wanted to determine which proteins change in expression in yeast chaperone mutants when the global ‘bias’ is eliminated, i.e. which proteins are differentially expressed above the constant up‐regulation caused by increases in cell size. As mentioned before, postacquisition normalisation is normally used for this purpose, and various procedures have been described in the literature, whose choice considerably affect the results and therefore downstream analysis 46. To determine the most appropriate normalisation method for our data we first assessed the effect of various normalisation procedures using yeast SILAC standards. To obtain the standards we mixed different amounts of heavy and light wild‐type yeast cells, processed the samples in the same way as the chaperone mutants, and acquired MS/MS data in technical triplicates. Standard A was a 1:1.6 heavy‐to‐light mix, and standard B was a 0.4:1 heavy‐to‐light mix giving two artificially skewed datasets. A successful normalisation will bring the median protein fold change to 0 while preserving quantitative differences between heavy and light samples. Normalised intensities of the datasets derived from these standards (data acquired in technical triplicates) should result in average fold change close to 0 (to account for the deliberate unequal mixing) and more importantly, very few significantly changing proteins (within an appropriate FDR cutoff) should be identified after statistical analysis. Inspection of protein ratio distributions, MA plots (Fig. 4 and Supporting Information Fig. 3) and Volcano plots (Supporting Information Fig. 3) pre‐ and post‐normalisation revealed that linear regression normalisation achieved this objective.

**Figure 5:**
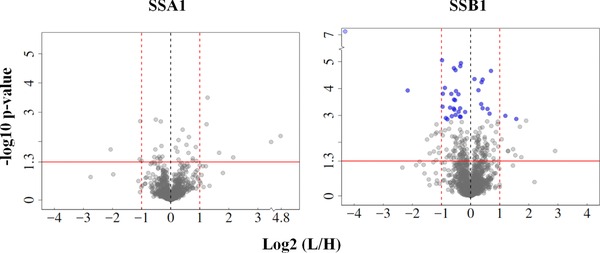
Volcano plots for the two Hsp70 yeast mutant proteomes compared to wild‐type yeast. Volcano plots show the normalised log_2_‐fold change and calculated ANOVA *p*‐value (–log_10_) for the *SSA1*, *SSB1*, chaperone mutants. Solid red line is at *p* value = 0.05 and points above it represent significantly changing proteins. The blue points represent proteins with FDR adjusted *p*‐value < 0.05 and dashed horizontal lines are positioned at log_2_ fold changes of –1 and 1, corresponding to two‐fold down/upregulation. Very few proteins exceed these cutoffs with statistical significance.

After linear regression normalisation, ANOVA statistics were calculated to determine the differentially regulated proteins between mutant and wild‐type yeast. Volcano plots showing the normalised log2 protein ratio and associated statistical significance measure (–log10 *p* value) for the two chaperone mutants are shown in Fig. 5. A full list of proteins along with log2(L/H ratios) pre‐ and postnormalisation, *p* values and adjusted *p‐*values and raw intensities from MaxQuant is provided in Supporting Information Table 1 in Supplementary Data. Based on replicate measurements and a standard ANOVA test, 103 ‘down’ and 86 ‘up’‐regulated proteins were identified as significantly changing in the *ΔSSB1* mutant and 15 ‘down’, 18 ‘up’ in *ΔSSA1* (*p* < 0.05). To control the FDR, *Benjamini–Hochberg* multiple testing correction 40 was applied, resulting in 27 down‐regulated proteins and 11 as up‐regulated for *ΔSSB1* at an adjusted *p*‐value threshold of 0.05. No proteins passed this threshold in the *ΔSSA1* mutant.

The most striking result of this quantitative analysis is the apparent absence of major proteome‐wide quantitative changes in the proteome upon deletion of an abundant *SSA1* chaperone and only minor changes above the global protein increase in the *SSB1* mutant strain. Although this is not particularly surprising in light of the modest phenotypic effects, in agreement with the original biochemical studies characterising SSA1 or SSB1 deletions (reviewed in 28), it is interesting from a molecular network perspective given the many interactions mediated by the two HSP70s. We next attempted to rationalise these effects by modelling the loss of the HSP70 in theoretical network calculations.

### 3.3 Chaperone mutant network analysis and workload calculations

In order to maintain cellular protein homeostasis, systems level adaptations in the chaperone network are likely to exist in the yeast mutants, beyond the simple redundancy expected from the SSA2 and SSB2 paralogues. We explored these potential mechanisms to account for the observed biological response to *SSA1* and *SSB1* gene deletions by examining the theoretical network structure in the mutants after removing the node in question and its attendant edges. We considered several network and node topology metrics 41, including network degree and node neighbourhood connectivity 47, shown in Supporting Information Fig. 4, and average clustering coefficient and BC, shown in Supporting Information Fig. 5.

We observe only modest changes to these metric distributions in the mutant networks. In Supporting Information Fig. 4A a small change (*p* < 0.05, D = 0.24, two sample Kolmogorov–Smirnov test) between neighbourhood connectivity distributions is observed when comparing the wild type and *SSB1* mutant networks, but this is the only significant difference. Hence, the network modelling suggests that, overall, the global chaperome is relatively unaffected by *SSA1* deletion and only modestly so by *SSB1* deletion.

At first glance, this might be surprising when considering network theory since these two HSP70s are major hubs of the chaperone network. However, despite this, even when deleting such a protein from a densely connected network, only a relatively limited number of nodes are affected. Other nodes retain their links and overall only a small average effect is observed and as a result, the chaperome maintains its characteristic scale‐free topology. The network is therefore robust in a topological sense and does not break down due to a single deletion. This is in agreement with our proteomics data where few significant changes in protein concentration are observed. Here, network topology as a whole provides a rationale, although not an immediate explanation for the experimental observations. All this is underlined by certain assumptions, the most important being that no major network re‐wiring takes place upon deletion. This is reasonable, since most SSA1/SSB1 interacting proteins are already linked to other HSP70 chaperones and, as noted before, not just the SSA2/SSB2 paralogues. In the case of SSA1 over 97% of its interactors are also linked to other chaperones and for SSB1 that number is 92%. In the light of this redundancy in the interactome it is likely that there is no need for re‐wiring, and the folding workload (considered explicitly later) is readily accommodated by the rest of the network.

After a general comparison of the global HSP70 mutant network properties, we next considered properties of the nodes (proteins) to rationalise emergent phenotypic properties of the chaperome system when compensating for the loss of a single element. We modelled the workload placed on individual nodes (chaperones) in the network, using two theoretical frameworks; BC and chaperone synthetic flux (referred to here as workload). The latter was defined in our previous work 8, 32, estimating the number of molecules per unit time passing through each chaperone en‐route to the native state. This estimate of chaperone workload predicted that SSB1 and SSA1 handle the biggest total substrate volume and total protein flux.

**Figure 6:**
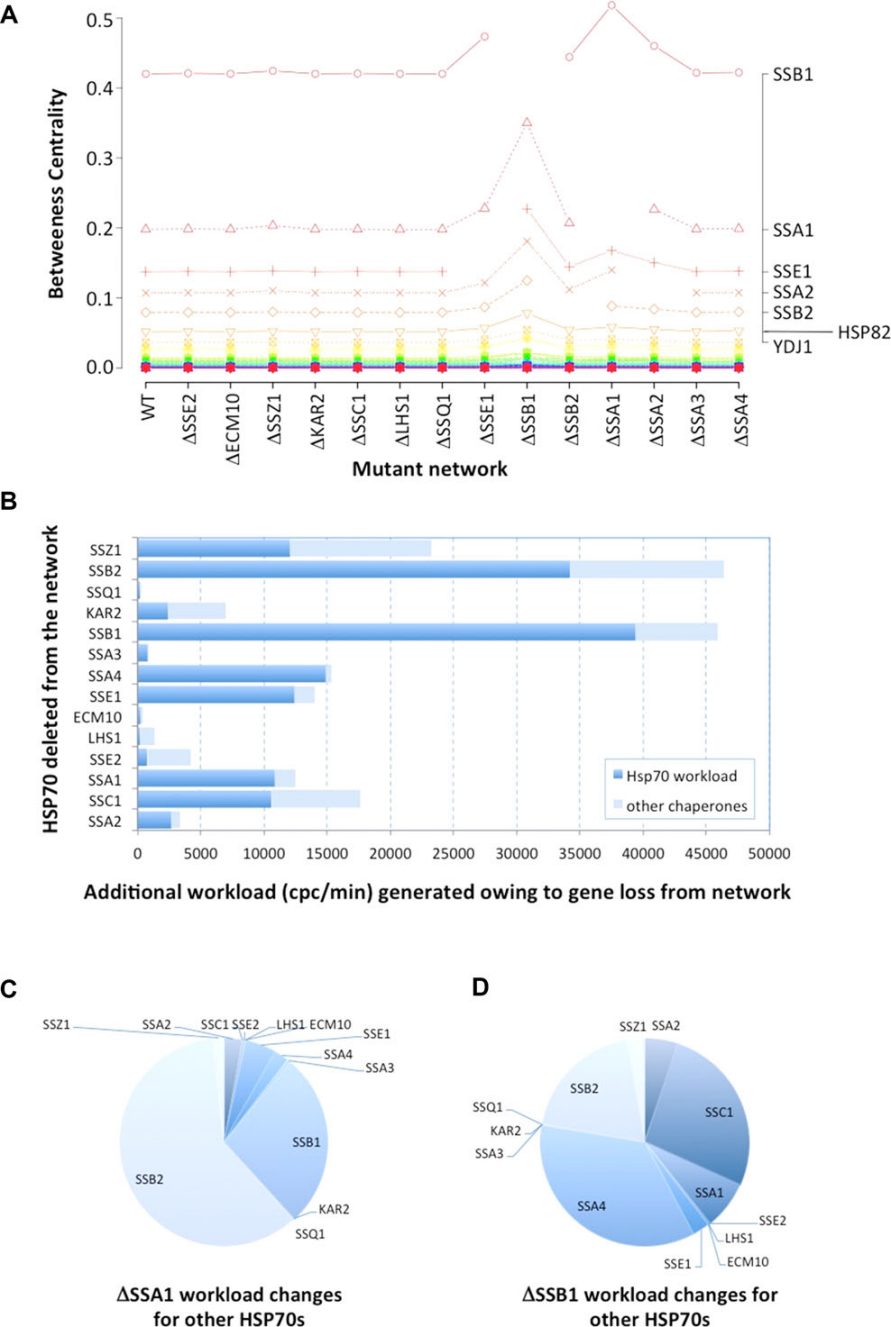
Betweeness centrality and chaperone workload for mutant strains. In (A), the BC values for the top 50 proteins in the wild‐type network are shown as well as their corresponding values in all theoretical HSP70 chaperone mutant networks. Each column in the figure corresponds to a different mutant. The left‐hand *y*‐axis gives the values for BC and right axis shows the names of top 7 (of 50) proteins ranked by BC. Large changes in BC for the top 7 proteins are visible in *SSA1* and *SSB1* mutant networks. (B) Barchart illustrating the total additional workload placed on other chaperones in the absence of a given HSP70 protein from the theoretical network. The estimated additional workload is expressed as copies per cell per minute (cpc/min) and split between HSP70s and other chaperones, when accommodating the absence of a given protein listed on the *y*‐axis. In panels (C) and (D), the additional workload split placed on the other HSP70s for the two chaperones considered here is shown as a pie chart, for the deletions of SSA1 and SSB1 in (C) and (D), respectively.

First, we considered BC. This is a network topology‐based metric, acting as a proxy for an individual chaperone's workload 48, 49, reflecting the ‘load’ or amount of ‘traffic’ a chaperone is exposed to. BC has also been applied to topological analysis of mammalian transcription networks where it was recognised to be the most representative parameter with regards to node biological significance 50. Notably, a clear positive correlation between the chaperone workload estimated previously 8, 32 and its BC exists (Spearman coefficient for top 15 chaperones = 0.89). Chaperones with higher BC index experience higher workloads, and could be regarded as functionally more important. Although it is a simplistic model of flux, BC can be calculated directly from the network topology and requires no additional knowledge of protein abundance or synthesis rates. We considered how the BC for selected HSP70s changes in yeast chaperone networks when other HSP70s are deleted. Figure 6A shows how BC varies for the top 50 proteins (ranked by BC value) in the wild type and mutant chaperone networks. The top 3 chaperones were found to have BC values of 0.42, 0.19, and 0.13 for SSB1, SSA1, and SSE1, respectively. When *SSB1* is deleted, SSA1, SSA2 and SSE1 display the biggest changes in BC, and in the absence of *SSA1* the BC values for SSB1, SSA2 and SSE1 notably increase. The results indicate those proteins become more prominent in the network and increase their topological control. Together with the fact that we did not detect any major changes in concentration of HSP70 proteins, these results suggest other HSP70 proteins collectively compensating for the loss of *SSA1/SSB1* function in the network by increasing their own workload, rather than unnecessarily increasing their concentrations. This mechanism is also consistent with known functions of HSP70 proteins, their redundancy and high substrate overlap.

In comparison to the network topological approach, we also estimated the attendant change in the folding workload placed on all other HSP70s when a single HSP70 is deleted from the genome. Workload was calculated using the same approach reported previously 8, 32 that presumes that in steady state the net change in protein concentration is zero, and hence we can estimate the synthesis rate of individual substrates using known protein half‐lives 51. Using protein abundances reported in PaxDB 13, the workload equates to a folding flux (number of molecules synthesized per min), which constitutes the work to be met by the chaperone pool. This is then assigned to the chaperones responsible for the folding of each substrate on a pro rata basis. When one chaperone is deleted from the network, additional effort must be shared out across the remaining chaperones. The sum of the attendant extra workload placed on the remaining chaperone pool is shown in Fig. 6B for HSP70 deletants. As can be seen, *SSB1* and *SSB2* loss results in the largest overall extra workload and *SSB1* deletion leads to the largest value for the other HSP70 chaperones, much larger than that attributable to a *SSA1* deletion. This is consistent with the observed perturbations in cell volume and differential protein expression in the two mutants, where *SSB1* elicits the greater effect, consistent with the additional stress we predict is placed on the chaperone network. The additional workload to be accommodated by specific HSP70s is also shown in Fig. 6c,d for the two mutants considered experimentally. Interestingly, the model predicts that proteins other than the closest paralogue picks up the most slack; e.g. SSB1/2 for the *SSA1* mutant and SSA4/SSC1 for the *SSB1* mutant, though SSB2 is also important in the latter case.

The data demonstrate the utility of the workload model to predict the major effect observed on *SSB1* deletion. However, it is also notable that there is not a perfect agreement between the workload and BC calculations, as the latter predicts a relatively modest affect on the HSP70 complement when *SSB2* and *SSA4* are deleted, but the workload calculations predict substantial extra work.

## 4 Concluding remarks

The strategy adopted here illustrates how quantitative proteomics, coupled to simple network modelling, can be used to observed changes in substrate protein levels in the chaperome and explain emergent properties of the system. The proteomics has revealed differences in total protein content between wild type and mutant strains for two exemplars, both at the global level (total cellular protein content) and local level (relative changes in individual protein concentrations). The global changes in protein amount can clearly be attributed to increased cell size of the mutant strains that we show is indeed the case from a relationship between cell size increase and normalisation scaling factor (Fig. 3C). However, interestingly, as the cell size increases, the relative protein concentration (for the majority of proteins) does not change. These results have implications for normalisation of proteomics in general, where users should consider their data carefully. Most normalisation strategies presume that there is no global change in the overall distribution of protein abundances, and detect statistically significant differences on this basis. However, for many reported yeast deletant strains there is clearly a global shift in protein amount, which in turn is placing additional demands on the proteostatic machinery of the cell.

The network analyses performed here illustrates their potential to describe and rationalise complex systems properties. Comparisons of network topology between WT and mutant strains show a relatively modest impact on most topological parameters, and hence we presume the networks do not change a lot. This is an interesting result as it is contrary to naïve expectation from a network perspective, which presumes that deletion of major hub proteins is deleterious and in some cases lethal. As is well known by yeast geneticists, this is clearly not the case for *SSA1* and *SSB1* 27, despite being the two biggest hubs identified by Gong et al. 7, and their deletion does not lead to apparent dramatic changes in relative protein concentrations. In fact, two other HSP70 chaperones (*SSC1* and *KAR2*) with fewer network connections are considered to be essential according to SGD 52. These general conclusions are matched by our workload calculations, which suggest that the additional workload caused by a deletion is buffered by the capacity remaining in the network. This functional redundancy in the yeast chaperone network may lead to changes in absolute chaperone levels that we were not able to observe via shotgun proteomic strategies. We intend to examine these effects in more detail, as well as under environmental stress, with targeted approaches. In parallel, further sophistications to the model can accommodate more realistic features such as sub‐cellular compartmentalisation to improve its predictive value.

## Supporting information

As a service to our authors and readers, this journal provides supporting information supplied by the authors. Such materials are peer reviewed and may be re‐organized for online delivery, but are not copy‐edited or typeset. Technical support issues arising from supporting information (other than missing files) should be addressed to the authors.

**Supplementary Figure S1. Example of representative peptides from ‘heavy’ yeast samples**. Good incorporation efficiency of Arg6 and Lys6 is visible. Two peptides from abundant yeast proteins and with high MS intensity are shown here; AVDDFIISIDGTANK ‐ Enolase 1 peptide and TANDVITIR from Pyruvate kinase 1. **RIA** = Intensity H/(Intensity H + Intensity L) *100%
**Supplementary Figure S2. Fold changes (from unnormalised log2 L/H ratios) of significantly down‐regulated proteins in *SSA1* and *SSB1* mutants**. Scatter plot shows unnormalised replicate measurements for each protein together with mean fold change and standard deviation error bars. Two proteins were negatively regulated in *SSA1* mutant and nine proteins in *SSB1*. In *ΔSSA1*, Long‐chain‐fatty‐acid‐CoA ligase 4 (LCF4; P47912) and UPF0743 protein YCR087C‐A (YC16; P37263) are significantly down‐regulated (ANOVA *p*‐value <0.05) with fold changes ‐1.9 and ‐0.3 respectively. While in *ΔSSB1* the following proteins showed statistically significant decreased expression: Glycine cleavage system H protein, mitochondrial (GCSH; P39726), fold change ‐3.9; 12 kDa heat shock protein (HSP12; P22943), fold change ‐ 1.7; Proteasome subunit alpha type‐2 (PSA2; P23639), fold change ‐ 1.1; Mitochondrial import inner membrane translocase subunit TIM13 (TIM13; P53299), fold change ‐0.6; ATP synthase subunit gamma, mitochondrial (ATPG; P38077), fold change ‐0.5; U6 snRNA‐associated Sm‐like protein LSm6 (LSM6; Q06406), fold change ‐0.5; Phosphomannomutase (PMM; P07283), fold change ‐0.5; Mitochondrial phosphate carrier protein (MPCP; P23641), fold change ‐0.5; and UPF0495 protein YPR010C‐A (YP010; A5Z2X5), fold change ‐0.3
**Supplementary Figure S3. Volcano plot showing the effect of linear regression normalisation on SILAC standards of unequal cell (protein) spike in**. Unnormalised protein ratios are skewed by unequal protein mixing. Successful normalisation ensures average fold change close to 0 and few significantly changing proteins (at appropriate FDR cutof), as differences within wild type yeast should not be large.
**Supplementary Figure S4. Distribution of topological parameters for the yeast chaperome networks**. Data are shown for the full chaperone network containing 4403 nodes and 21946 undirected interactions. a) Neighbourhood connectivity distribution describes the average degree (connectivity) of all neighbours of a protein with a given degree. Here, the disassortative behaviour of the network is visible; on average, nodes with low degree (chaperone client proteins in most cases) connect to nodes of high degree (chaperone hubs). The distribution of neighbourhood connectivity values for WT and mutant networks is described by an empirical cumulative distribution function and the K‐S test is used to compute the D statistic (highest deviation between the distributions). The distribution of *SSB1* mutant network differs significantly from the WT distribution. b) Average degree (k) distribution shows the probability that a protein has a degree larger or equal to k.
**Supplementary Figure S5. Distribution of additional topological parameters for the yeast chaperome network**. Clustering coefficient of a protein *n* quantifies what fraction of possible connections between all neighbours of *n* passes through it. Betweenness centrality describes how central a node is in the network and what influence it has over the transfer of information through the network. Both these parameters are plotted here as a function of node degree.

Table 1

## References

[1] Kim, Y. E. , Hipp, M. S. , Bracher, A. , Hayer‐Hartl, M. , Hartl, F. U. , Molecular chaperone functions in protein folding and proteostasis. Ann. Rev. Biochem. 2013, 82, 323–355. 23746257 10.1146/annurev-biochem-060208-092442

[2] Vabulas, R. M. , Raychaudhuri, S. , Hayer‐Hartl, M. , Hartl, F. U. , Protein folding in the cytoplasm and the heat shock response. Cold Spring Harbor PerspectivesBiol. 2010, 2, a004390. 10.1101/cshperspect.a004390 PMC2982175 21123396

[3] Morano, K. A. , Grant, C. M. , Moye‐Rowley, W. S. , The response to heat shock and oxidative stress in *Saccharomyces cerevisiae* . Genetics 2012, 190, 1157–1195. 22209905 10.1534/genetics.111.128033 PMC3316637

[4] Hartl, F. U. , Hayer‐Hartl, M. , Protein folding—molecular chaperones in the cytosol: from nascent chain to folded protein. Science 2002, 295, 1852–1858. 11884745 10.1126/science.1068408

[5] Ferrell, J. E., Jr. , Q&A: systems biology. J. Biol. 2009, 8, 2–2. 19222866 10.1186/jbiol107 PMC2656213

[6] Bhalla, U. S. , Iyengar, R. , Emergent properties of networks of biological signaling pathways. Science 1999, 283, 381–387. 9888852 10.1126/science.283.5400.381

[7] Gong, Y. , Kakihara, Y. , Krogan, N. , Greenblatt, J. et al., An atlas of chaperone‐protein interactions in *Saccharomyces cerevisiae*: implications to protein folding pathways in the cell. Mol. Syst. Biol. 2009, 5, 275. 19536198 10.1038/msb.2009.26 PMC2710862

[8] Lawless, C. , Hubbard, S. J. , in: Houry W. A. (Ed.), The Molecular Chaperones Interaction Networks in Protein Folding and Degradation, Springer 2014.

[9] Bogumil, D. , Landan, G. , Ilhan, J. , Dagan, T. , Chaperones divide yeast proteins into classes of expression level and evolutionary rate. Genome Biol. Evol. 2012, 4, 618–625. 22417914 10.1093/gbe/evs025 PMC3381671

[10] Saibil, H. , Chaperone machines for protein folding, unfolding and disaggregation. Nat. Rev. Mol. Cell Biol. 2013, 14, 630–642. 24026055 10.1038/nrm3658 PMC4340576

[11] Hartl, F. U. , Bracher, A. , Hayer‐Hartl, M. , Molecular chaperones in protein folding and proteostasis. Nature 2011, 475, 324–332. 21776078 10.1038/nature10317

[12] Kampinga, H. H. , Craig, E. A. , ‘The HSP70 chaperone machinery: J proteins as drivers of functional specificity. Nat. Rev. Mol. Cell Biol. 2010, 11, 579–592. 20651708 10.1038/nrm2941 PMC3003299

[13] Wang, M. , Weiss, M. , Simonovic, M. , Haertinger, G. et al., PaxDb, a database of protein abundance averages across all three domains of life. Mol. Cell. Proteomics 2012, 11, 492–500. 22535208 10.1074/mcp.O111.014704 PMC3412977

[14] Boorstein, W. R. , Ziegelhoffer, T. , Craig, E. A. , Molecular evolution of the Hsp70 multigene family. J. Mol. Evol. 1994, 38, 1–17. 8151709 10.1007/BF00175490

[15] Kominek, J. , Marszalek, J. , Neuveglise, C. , Craig, E. A. , Williams, B. L. , The complex evolutionary dynamics of Hsp70s: a genomic and functional perspective. Genome Biol. Evol. 2013, 5, 2460–2477. 24277689 10.1093/gbe/evt192 PMC3879978

[16] Daugaard, M. , Rohde, M. , Jaattela, M. , The heat shock protein 70 family: highly homologous proteins with overlapping and distinct functions. Febs. Lett. 2007, 581, 3702–3710. 17544402 10.1016/j.febslet.2007.05.039

[17] Wernerwashburne, M. , Stone, D. E. , Craig, E. A. , Complex interactions among members of an essential subfamily of Hsp70 genes in *Saccharomyces cerevisiae* . Mol. Cell. Biol. 1987, 7, 2568–2577. 3302682 10.1128/mcb.7.7.2568-2577.1987 PMC365392

[18] Becker, J. , Craig, E. A. , Heat‐shock proteins as molecular chaperones. Eur. J. Biochem. 1994, 219, 11–23. 8306977 10.1007/978-3-642-79502-2_2

[19] Pfund, C. , Huang, P. , Lopez‐Hoyo, N. , Craig, E. A. , Divergent functional properties of the ribosome‐associated molecular chaperone Ssb compared with other Hsp70s. Mol. Biol. Cell 2001, 12, 3773–3782. 11739779 10.1091/mbc.12.12.3773 PMC60754

[20] Lopez, N. , Halladay, J. , Walter, W. , Craig, E. A. , SSB, encoding a ribosome‐associated chaperone, is coordinately regulated with ribosomal protein genes. J. Bacteriol. 1999, 181, 3136–3143. 10322015 10.1128/jb.181.10.3136-3143.1999 PMC93769

[21] Nelson, R. J. , Ziegelhoffer, T. , Nicolet, C. , Wernerwashburne, M. , Craig, E. A. , The translation machinery and 70 KD heat‐shock protein cooperate in protein‐synthesis. Cell 1992, 71, 97–105. 1394434 10.1016/0092-8674(92)90269-i

[22] Craig, E. A. , Jacobsen, K. , Mutations of the heat inducible 70‐kilodalton genes of yeast confer temperature sensitive growth. Cell 1984, 38, 841–849. 6386178 10.1016/0092-8674(84)90279-4

[23] Craig, E. A. , Jacobsen, K. , Mutations in cognate genes of *Saccharomyces cerevisiae* hsp70 result in reduced growth‐rates at low‐temperatures. Mol. Cell. Biol. 1985, 5, 3517–3524. 3915778 10.1128/mcb.5.12.3517-3524.1985 PMC369182

[24] Young, M. R. , Craig, E. A. , *Saccharomyces cerevisiae* Hsp70 heat‐shock elements are functionally distinct. Mol. Cell. Biol. 1993, 13, 5637–5646. 8355706 10.1128/mcb.13.9.5637-5646.1993 PMC360292

[25] Bukau, B. , Horwich, A. L. , The Hsp70 and Hsp60 chaperone machines. Cell 1998, 92, 351–366. 9476895 10.1016/s0092-8674(00)80928-9

[26] Ellwood, M. S. , Craig, E. A. , Differential regulation of the 70K heat‐shock gene and related genes in *Saccharomyces cerevisiae* . Mol. Cell. Biol. 1984, 4, 1454–1459. 6436685 10.1128/mcb.4.8.1454-1459.1984 PMC368934

[27] Craig, E. A. , Ziegelhoffer, T. , Nelson, J. , Laloraya, S. , and Halladay, J. , Cold Spring Harbor Symposia on Quantitative Biology: Protein Kinesis: The Dynamics of Protein Trafficking and Stability, Cold Spring Harbor Laboratory, NY 1995, pp. 441–449. 10.1101/sqb.1995.060.01.049 8824418

[28] Kabani, M. , Martineau, C. N. , Multiple Hsp70 isoforms in the eukaryotic cytosol: mere redundancy or functional specificity? Curr. Genomics 2008, 9, 338–348. 19471609 10.2174/138920208785133280 PMC2685646

[29] Ingolia, T. D. , Slater, M. R. , Craig, E. A. , *Saccharomyces cerevisiae* contains a complex multigene family related to the major heat shock‐inducible gene of drosophila. Mol. Cell. Biol. 1982, 2, 1388–1398. 6761581 10.1128/mcb.2.11.1388-1398.1982 PMC369943

[30] Pfund, C. , Lopez‐Hoyo, N. , Ziegelhoffer, T. , Schilke, B. A. et al., The molecular chaperone Ssb from *Saccharomyces cerevisiae* is a component of the ribosome nascent chain complex. Embo. J. 1998, 17, 3981–3989. 9670014 10.1093/emboj/17.14.3981 PMC1170732

[31] Gao, B. C. , Biosca, J. , Craig, E. A. , Greene, L. E. , Eisenberg, E. , Uncoating of coated vesicles by yeast hsp70 proteins. J. Biol. Chem. 1991, 266, 19565–19571. 1833403

[32] Philip Brownridge, C. L. , Payapilly, A. B. , Lanthaler, K. , Holman, S. W. et al. Quantitative analysis of chaperone network throughput in budding yeast. Proteomics 2013, 13, 1276–1291. 23420633 10.1002/pmic.201200412 PMC3791555

[33] Ong, S. E. , Blagoev, B. , Kratchmarova, I. , Kristensen, D. B. et al., Stable isotope labeling by amino acids in cell culture, SILAC, as a simple and accurate approach to expression proteomics. Mol. Cell. Proteomics 2002, 1, 376–386. 12118079 10.1074/mcp.m200025-mcp200

[34] Cox, J. , Mann, M. , MaxQuant enables high peptide identification rates, individualized p.p.b.‐range mass accuracies and proteome‐wide protein quantification. Nat. Biotechnol. 2008, 26, 1367–1372. 19029910 10.1038/nbt.1511

[35] Cox, J. , Matic, I. , Hilger, M. , Nagaraj, N. et al., A practical guide to the MaxQuant computational platform for SILAC‐based quantitative proteomics. Nat. Protocols 2009, 4, 698–705. 19373234 10.1038/nprot.2009.36

[36] Cox, J. , Neuhauser, N. , Michalski, A. , Scheltema, R. A. et al., Andromeda: a peptide search engine integrated into the MaxQuant environment. J. Proteome Res. 2011, 10, 1794–1805. 21254760 10.1021/pr101065j

[37] RDevelopmentCoreTeam , R: A Language and Environment for Statistical Computing 2013.

[38] Taverner, T. , Karpievitch, Y. V. , Polpitiya, A. D. , Brown, J. N. et al., DanteR: an extensible R‐based tool for quantitative analysis of ‐omics data. Bioinformatics 2012, 28, 2404–2406. 22815360 10.1093/bioinformatics/bts449 PMC3436848

[39] Chambers, J. M. , in: Hastie J. M. C. a. T. J. (Ed.), Statistical Models in S, Wadsworth & Brooks/Cole, Pacific Grove, CA 1992.

[40] Noble, W. S. , How does multiple testing correction work? Nat. Biotechnol. 2009, 27, 1135–1137. 20010596 10.1038/nbt1209-1135 PMC2907892

[41] Shannon, P. , Markiel, A. , Ozier, O. , Baliga, N. S. et al., Cytoscape: a software environment for integrated models of biomolecular interaction networks. Genome Res. 2003, 13, 2498–2504. 14597658 10.1101/gr.1239303 PMC403769

[42] Assenov, Y. , Ramirez, F. , Schelhorn, S.‐E. , Lengauer, T. , Albrecht, M. , Computing topological parameters of biological networks. Bioinformatics 2008, 24, 282–284. 18006545 10.1093/bioinformatics/btm554

[43] George Marsaglia, W. W. T. , Wang, J. , Evaluating Kolmogorov's distribution. J. Stat. Software 2003, 8, 1–4.

[44] de Godoy, L. M. F. , Olsen, J. V. , Cox, J. , Nielsen, M. L. et al., Comprehensive mass‐spectrometry‐based proteome quantification of haploid versus diploid yeast. Nature 2008, 455, U1251–U1260. 10.1038/nature07341 18820680

[45] Saito, T. L. , Ohtani, M. , Sawai, H. , Sano, F. et al., SCMD: *Saccharomyces cerevisiae* morphological database. Nucleic Acids Res. 2004, 32, D319–D322. 14681423 10.1093/nar/gkh113 PMC308847

[46] Kultima, K. , Nilsson, A. , Scholz, B. , Rossbach, U. L. et al., Development and evaluation of normalization methods for label‐free relative quantification of endogenous peptides. Mol. Cell. Proteomics 2009, 8, 2285–2295. 19596695 10.1074/mcp.M800514-MCP200 PMC2758756

[47] Maslov, S. , Sneppen, K. , Specificity and stability in topology of protein networks. Science 2002, 296, 910–913. 11988575 10.1126/science.1065103

[48] Freeman, L. C. , Set of measures of centrality based on betweenness. Sociometry 1977, 40, 35–41.

[49] Brandes, U. , A faster algorithm for betweenness centrality. J. Math. Sociol. 2001, 25, 163–177.

[50] Potapov, A. P. , Voss, N. , Sasse, N. , Wingender, E. , Topology of mammalian transcription networks. Genome Inform. Int. Conf. Genome Informatics 2005, 16, 270–278. 16901109

[51] Belle, A. , Tanay, A. , Bitincka, L. , Shamir, R. , O'Shea, E. K. , Quantification of protein half‐lives in the budding yeast proteome. Proc. Nat. Acad. Sci. USA 2006, 103, 13004–13009. 16916930 10.1073/pnas.0605420103 PMC1550773

[52] Cherry, J. M. , Hong, E. L. , Amundsen, C. , Balakrishnan, R. et al., Saccharomyces genome database: the genomics resource of budding yeast. Nucleic Acids Res. 2012, 40, D700–D705. 22110037 10.1093/nar/gkr1029 PMC3245034

