# Supplementary material for: Quantitative proteomics and network analysis of SSA1 and SSB1 deletion mutants reveals robustness of chaperone HSP70 network in Saccharomyces cerevisiae
Source: Proteomics. 2015 Apr 10;15(18):3126–39. doi: 10.1002/pmic.201400527 (PMC4979674; doi:10.1002/pmic.201400527)
Supplement: Supplementary file 1 — Supplementary Figure S1. Example of representative peptides from ‘heavy’ yeast samples. Good incorporation efficiency of Arg6 and Lys6 is visible. Two peptides from abundant yeast proteins and with high MS intensity are shown here; AVDDFIISIDGTANK ‐ Enolase 1 peptide and TANDVITIR from Pyruvate kinase 1. RIA = Intensity H/(Intensity H + Intensity L) *100% Supplementary Figure S2. Fold changes (from unnormalised log2 L/H ratios) of significantly down‐regulated proteins in SSA1 and SSB1 mutants. Scatter plot shows unnormalised replicate measurements for each protein together with mean fold change and standard deviation error bars. Two proteins were negatively regulated in SSA1 mutant and nine proteins in SSB1. In ΔSSA1, Long‐chain‐fatty‐acid‐CoA ligase 4 (LCF4; P47912) and UPF0743 protein YCR087C‐A (YC16; P37263) are significantly down‐regulated (ANOVA p‐value <0.05) with fold changes ‐1.9 and ‐0.3 respectively. While in ΔSSB1 the following proteins showed statistically significant decreased expression: Glycine cleavage system H protein, mitochondrial (GCSH; P39726), fold change ‐3.9; 12 kDa heat shock protein (HSP12; P22943), fold change ‐ 1.7; Proteasome subunit alpha type‐2 (PSA2; P23639), fold change ‐ 1.1; Mitochondrial import inner membrane translocase subunit TIM13 (TIM13; P53299), fold change ‐0.6; ATP synthase subunit gamma, mitochondrial (ATPG; P38077), fold change ‐0.5; U6 snRNA‐associated Sm‐like protein LSm6 (LSM6; Q06406), fold change ‐0.5; Phosphomannomutase (PMM; P07283), fold change ‐0.5; Mitochondrial phosphate carrier protein (MPCP; P23641), fold change ‐0.5; and UPF0495 protein YPR010C‐A (YP010; A5Z2X5), fold change ‐0.3 Supplementary Figure S3. Volcano plot showing the effect of linear regression normalisation on SILAC standards of unequal cell (protein) spike in. Unnormalised protein ratios are skewed by unequal protein mixing. Successful normalisation ensures average fold change close to 0 and few significantly changing proteins (at appropriate [file PMIC-15-3126-s001.docx]

Quantitative proteomics and network analysis of SSA1 and SSB1 deletion mutants reveals robustness of chaperone HSP70 network in *Saccharomyces cerevisiae*

**SUPPLEMENTARY MATERIALS**

Andrew Jarnuczak^1^, Claire E. Eyers^2^, Jean-Marc Schwartz^1^, Christopher M. Grant^1^, Simon J. Hubbard^1^

1. Faculty of Life Sciences, Michael Smith Building, Oxford Road, Manchester, M13 9PT

2. Centre for Proteome Research, Department of Biochemistry, Institute of Integrative Biology, University of Liverpool, Liverpool, L69 7ZB, UK

Address for correspondence: Prof Simon Hubbard

Faculty of Life Sciences

University of Manchester

Michael Smith Building, Oxford Road

Manchester M13 9PT

UK

Email: simon.hubbard@manchester.ac.uk

Tel: +44 (0)161 306 8930

**Supplementary Figures Legends**

**Supplementary Figure S1. Example of representative peptides from ‘heavy’ yeast samples**. Good incorporation efficiency of Arg6 and Lys6 is visible. Two peptides from abundant yeast proteins and with high MS intensity are shown here; AVDDFIISIDGTANK - Enolase 1 peptide and TANDVITIR from Pyruvate kinase 1. **RIA** = Intensity H/(Intensity H + Intensity L) *100%

**Supplementary Figure S2. Fold changes (from unnormalised log2 L/H ratios) of significantly down-regulated proteins in *SSA1* and *SSB1* mutants.** Scatter plot shows unnormalised replicate measurements for each protein together with mean fold change and standard deviation error bars. Two proteins were negatively regulated in *SSA1* mutant and nine proteins in *SSB1*. In *ΔSSA1*, Long-chain-fatty-acid-CoA ligase 4 (LCF4; P47912) and UPF0743 protein YCR087C-A (YC16; P37263) are significantly down-regulated (ANOVA p-value <0.05) with fold changes -1.9 and -0.3 respectively. While in *ΔSSB1* the following proteins showed statistically significant decreased expression: Glycine cleavage system H protein, mitochondrial (GCSH; P39726), fold change -3.9; 12 kDa heat shock protein (HSP12; P22943), fold change - 1.7; Proteasome subunit alpha type-2 (PSA2; P23639), fold change - 1.1; Mitochondrial import inner membrane translocase subunit TIM13 (TIM13; P53299), fold change -0.6; ATP synthase subunit gamma, mitochondrial (ATPG; P38077), fold change -0.5; U6 snRNA-associated Sm-like protein LSm6 (LSM6; Q06406), fold change -0.5; Phosphomannomutase (PMM; P07283), fold change -0.5; Mitochondrial phosphate carrier protein (MPCP; P23641), fold change -0.5; and UPF0495 protein YPR010C-A (YP010; A5Z2X5), fold change -0.3

**Supplementary Figure S3. Volcano plot showing the effect of linear regression normalisation on SILAC standards of unequal cell (protein) spike in**. Unnormalised protein ratios are skewed by unequal protein mixing. Successful normalisation ensures average fold change close to 0 and few significantly changing proteins (at appropriate FDR cutof), as differences within wild type yeast should not be large.

**Supplementary Figure S4. Distribution of topological parameters for the yeast chaperome networks**. Data are shown for the full chaperone network containing 4403 nodes and 21946 undirected interactions. a) Neighbourhood connectivity distribution describes the average degree (connectivity) of all neighbours of a protein with a given degree. Here, the disassortative behaviour of the network is visible; on average, nodes with low degree (chaperone client proteins in most cases) connect to nodes of high degree (chaperone hubs). The distribution of neighbourhood connectivity values for WT and mutant networks is described by an empirical cumulative distribution function and the K-S test is used to compute the D statistic (highest deviation between the distributions). The distribution of *SSB1* mutant network differs significantly from the WT distribution. b) Average degree (k) distribution shows the probability that a protein has a degree larger or equal to k.

**Supplementary Figure S5. Distribution of additional topological parameters for the yeast chaperome network**. Clustering coefficient of a protein *n* quantifies what fraction of possible connections between all neighbours of *n* passes through it. Betweenness centrality describes how central a node is in the network and what influence it has over the transfer of information through the network. Both these parameters are plotted here as a function of node degree.

**Supplementary Figure S1**


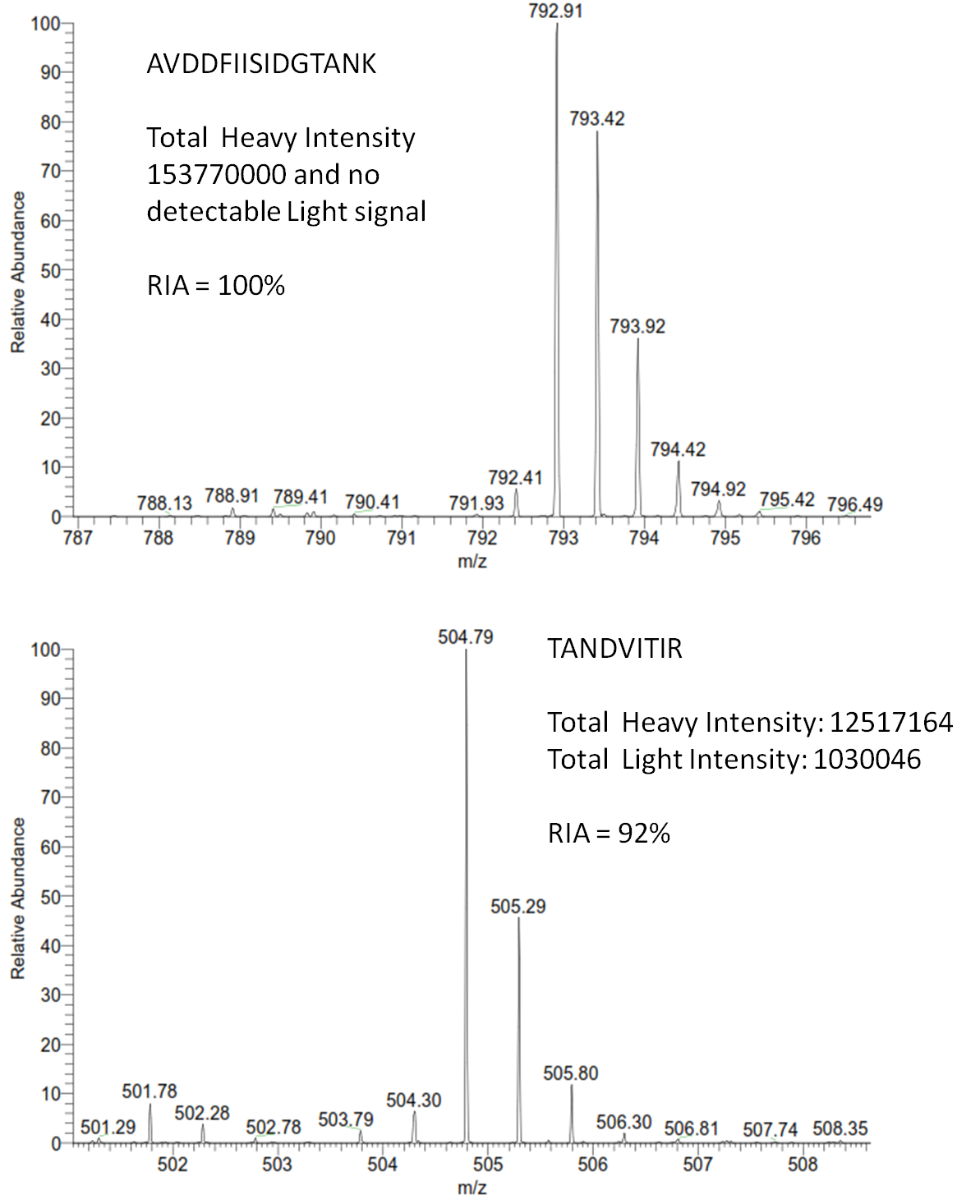


**Supplementary Figure 2**


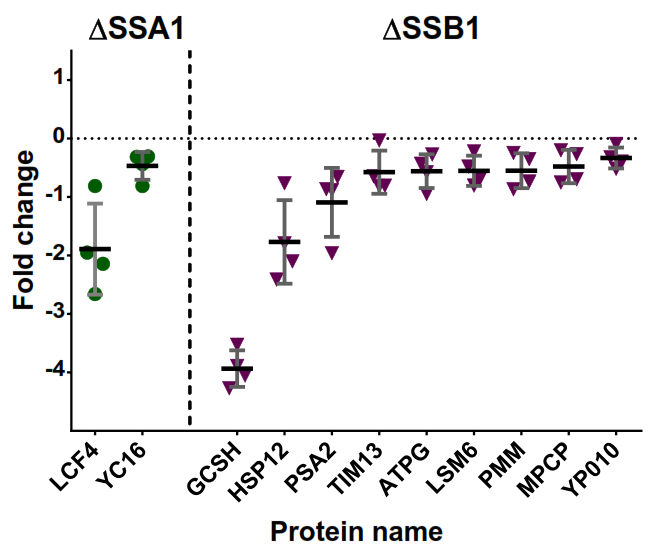


**Supplementary Figure S3**

**
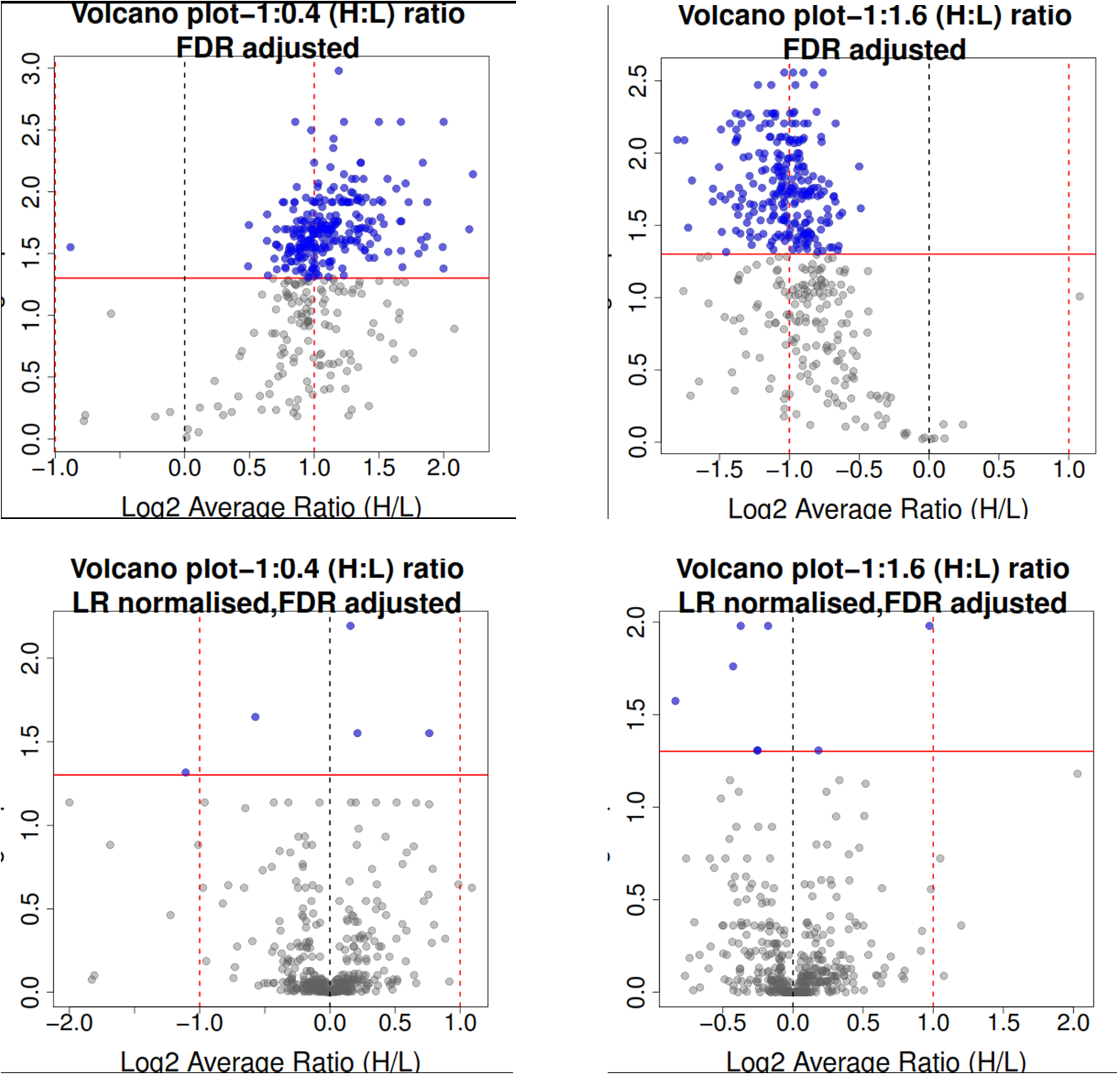
**

**Supplementary Figure S4**

**
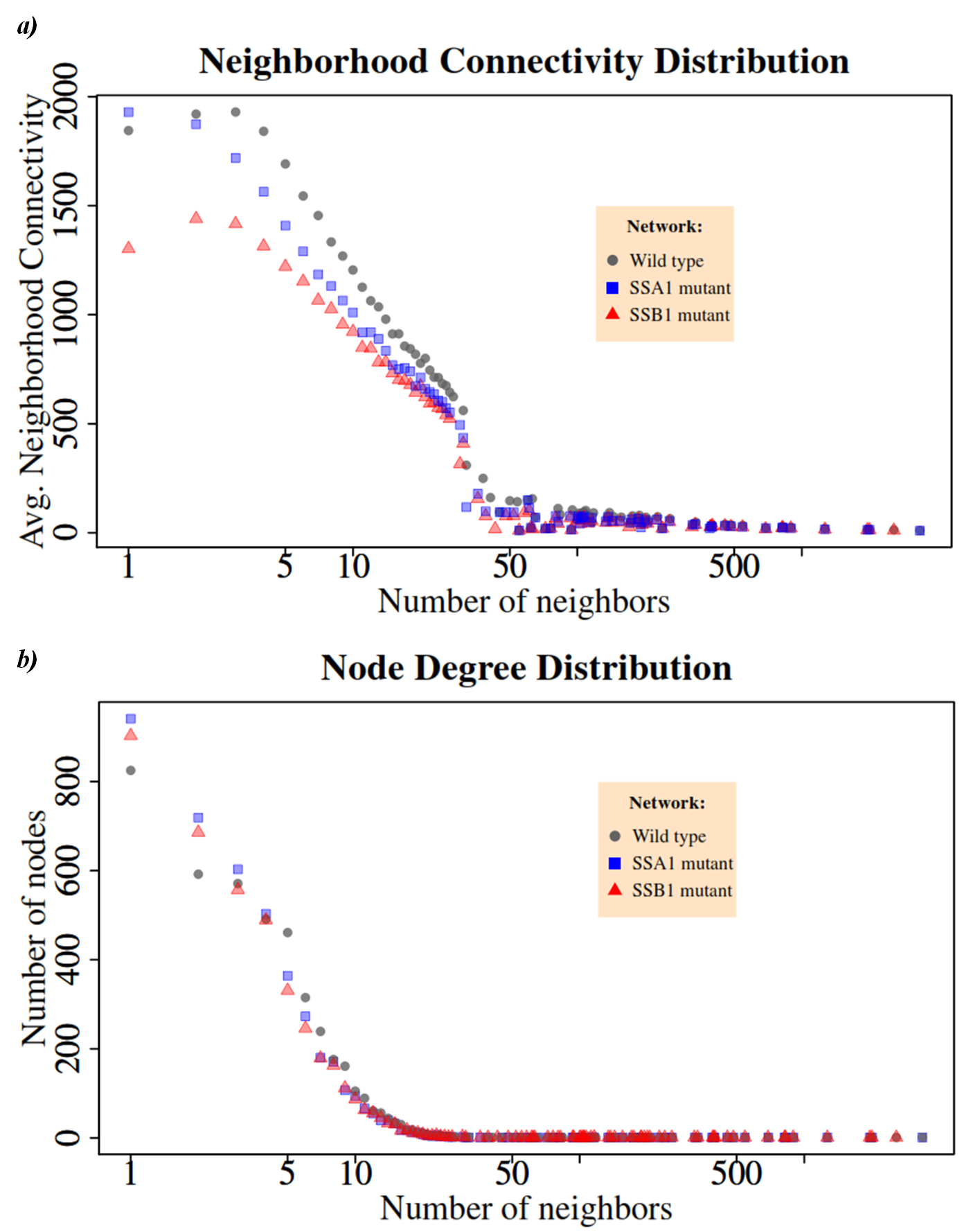
**

**Supplementary Figure S5**

**
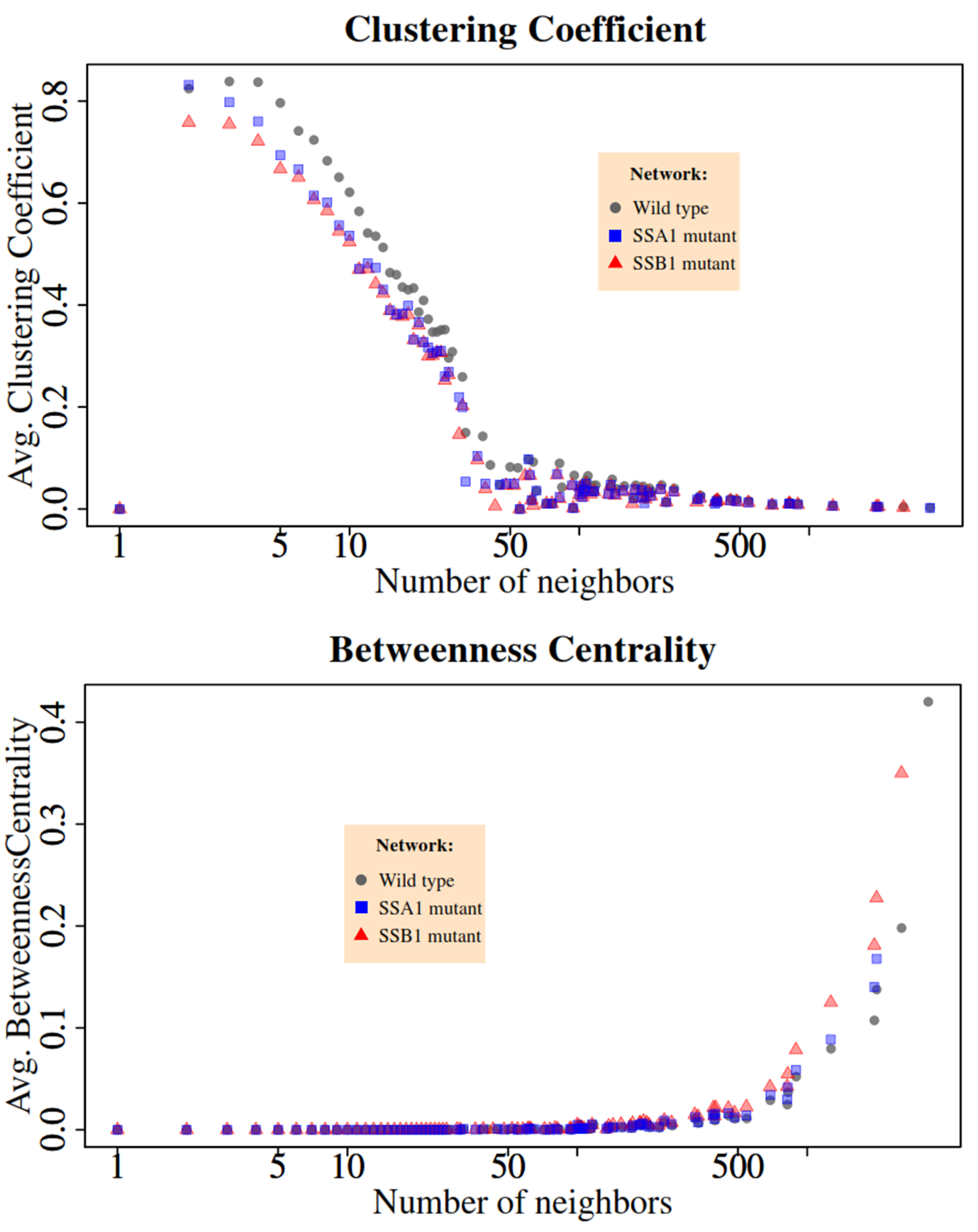
**
